# Effects of prone positioning on ARDS outcomes of trauma and surgical patients: a systematic review and meta-analysis

**DOI:** 10.1186/s12890-023-02805-w

**Published:** 2023-12-13

**Authors:** Vorakamol Phoophiboon, Natthida Owattanapanich, Weerapat Owattanapanich, Morgan Schellenberg

**Affiliations:** 1 Division of Critical Care Medicine, Department of Medicine, Faculty of Medicine, Chulalongkorn University, Bangkok, Thailand; 2 Excellence Center for Critical Care Medicine, King Chulalongkorn Memorial Hospital, Thai Red Cross Society, Bangkok, Thailand; 3 Department of Critical Care Medicine, St. Michael’s Hospital, Unity Health Toronto, University of Toronto, Toronto, Canada; 4 Division of Trauma Surgery, Department of Surgery, Faculty of Medicine Siriraj Hospital, Mahidol University, Bangkok, Thailand; 5 Division of Hematology, Department of Medicine, Faculty of Medicine Siriraj Hospital, Mahidol University, Bangkok, Thailand; 6 Trauma and Surgical Critical Care, LAC+USC Medical Center, University of Southern California, Los Angeles, CA USA

**Keywords:** ARDS, ICU, Prone positioning, Surgery, Trauma

## Abstract

**Background:**

Prone position is an option for rescue therapy for acute respiratory distress syndrome. However, there are limited relevant data among trauma and surgical patients, who may be at increased risk for complications following position changes. This study aimed to identify the benefits and risks of proning in this patient subgroup.

**Methods:**

Follow the PRISMA 2020, MEDLINE and EMBASE database searches were conducted. Additional search of relevant primary literature and review articles was also performed. A random effects model was used to estimate the PF ratio, mortality rate, mechanical ventilator days, and intensive care unit length of stay using Review Manager 5.4.1 software.

**Results:**

Of 1,128 studies, 15 articles were included in this meta-analysis. The prone position significantly improved the PF ratio compared with the supine position (mean difference, 79.26; 95% CI, 53.38 to 105.13). The prone position group had a statistically significant mortality benefit (risk ratio [RR], 0.48; 95% CI, 0.35 to 0.67). Although there was no significant difference in the intensive care unit length of stay, the prone position significantly decreased mechanical ventilator days (-2.59; 95% CI, -4.21 to -0.97). On systematic review, minor complications were frequent, especially facial edema. There were no differences in local wound complications.

**Conclusions:**

The prone position has comparable complications to the supine position. With its benefits of increased oxygenation and decreased mortality, the prone position can be considered for trauma and surgical patients. A prospective multicenter study is warranted.

## Background

Regarding the Berlin definition 2012, acute respiratory distress syndrome (ARDS) is an acute respiratory failure with bilateral opacities which is not fully explained by cardiac failure or fluid overload. It has been classified into 3 severities based on the ratio of arterial oxygen partial pressure to fractional inspired oxygen (PF ratio) with PEEP or CPAP at least 5 cmH_2_O [1]. In the LUNG SAFE study [2], the largest observational ARDS study, the geographic variation in ARDS incidence ranged from 0.27 to 0.57 cases per intensive care unit (ICU) bed per 4 weeks, comprising 10% of ICU admissions. The primary diagnosis among most ARDS patients in this study was medical or surgical (65%), with trauma patients comprising 46% of over one hundred surgical patients included.

Prone positioning is theoretically helpful in ARDS by increasing homogeneous ventilation distribution, modifying chest wall and lung compliance/elastance, reducing ventilator-induced lung injury (VILI) and facilitating secretion mobilization and clearance [3]. PROSEVA trial, a large multi-center randomized control study, demonstrated that early application of prolonged prone-positioning significantly decreased mortality among severe ARDS patients [4]. However, it must be noted that studies on the benefits of prone positioning in ARDS have been performed primarily among medical patients (79–89%), with surgical patients making up only 4–9% and trauma patients comprising only 2–7% of all patients examined [4, 5]. Because trauma and surgical patients may be at increased risk from position changes as compared to medical patients, it is possible that the balance of risks and benefits for prone positioning in ARDS are different in this specific subset of patients.

Because of the limited evidence of the use of prone positioning among ARDS patients specifically among trauma or surgical patients, we endeavoured to determine the effect of prone positioning towards ARDS outcomes (P/F ratio - the ratio of arterial oxygen partial pressure to fractional inspired oxygen, mortality, ICU length of stay and mechanical ventilator day) among trauma and surgical patients.

## Methods

### Eligibility criteria

This systematic review and meta-analysis followed the Preferred Reporting Items for Systematic Reviews and Meta-analysis (PRISMA) [6]. We applied the following inclusion criteria: trials studying the prone position and acute respiratory distress syndrome and trauma-surgical patient. All experimental and cohort studies, whether retrospective or prospective, were included. Exclusion criteria were as follows: case report, case series and ongoing trials. The outcome measures were P/F ratio, mortality, ICU length of stay and mechanical ventilator days.

### Information sources and search strategy

Electronic systematic searches were conducted for articles published before November 30, 2022, using MEDLINE and EMBASE. The medical subject headings used in our searches were “trauma,” “surgical,” “acute respiratory failure,” and “prone position.” There was no language restriction.

### Study selection and data collection

Two investigators (V.P. and N.O.) independently selected prospective studies using the predetermined eligibility criteria by title and abstract screening. The references of the included studies were also manually reviewed for additional relevant studies. Disagreements relating to any aspect of the data extraction process were resolved through discussion with the third investigator (W.O.), with the final decision made by consensus. The full-text articles of the selected studies were reviewed for the final study selection. The data were extracted and analyzed from the included studies.

### Characteristics of included studies

The studies included in this meta-analysis were cohort or experimental studies (prospective or retrospective) of trauma or surgical patients diagnosed with ARDS.

### Quality assessment

Regarding Cochrane recommendation, two investigators (V.P. and N.O.) assessed the quality of each study using the Cochrane risk-of bias tool for randomized trials (RoB 2) and the Risk Of Bias In Non-randomized Studies of Interventions (ROBINS-I) for non-randomised studies [7, 8].

### Statistical analysis

All statistical analyses were performed using Review Manager 5.4.1 software from the Cochrane Collaboration (London, United Kingdom). We extracted the proportions and 95% confidence intervals (CIs) from each study and pooled them using the random effect model. Cochran’s Q test was performed and quantified using the I^2^ statistic to determine the statistical heterogeneity among the included studies. An I^2^ value of 0–25% represents insignificant heterogeneity, greater than 25% but less than or equal to 50% represents low heterogeneity, greater than 50% but less than or equal to 75% represents moderate heterogeneity, and greater than 75% represents high heterogeneity. *P*-value less than 0.05 were considered statistically significant. The presence of a publication bias was visualized by a funnel plot. This study was registered at as http://www.inplasy.com as #INPLASY202330102.

## Results

### Search results

**Fig. 1:**
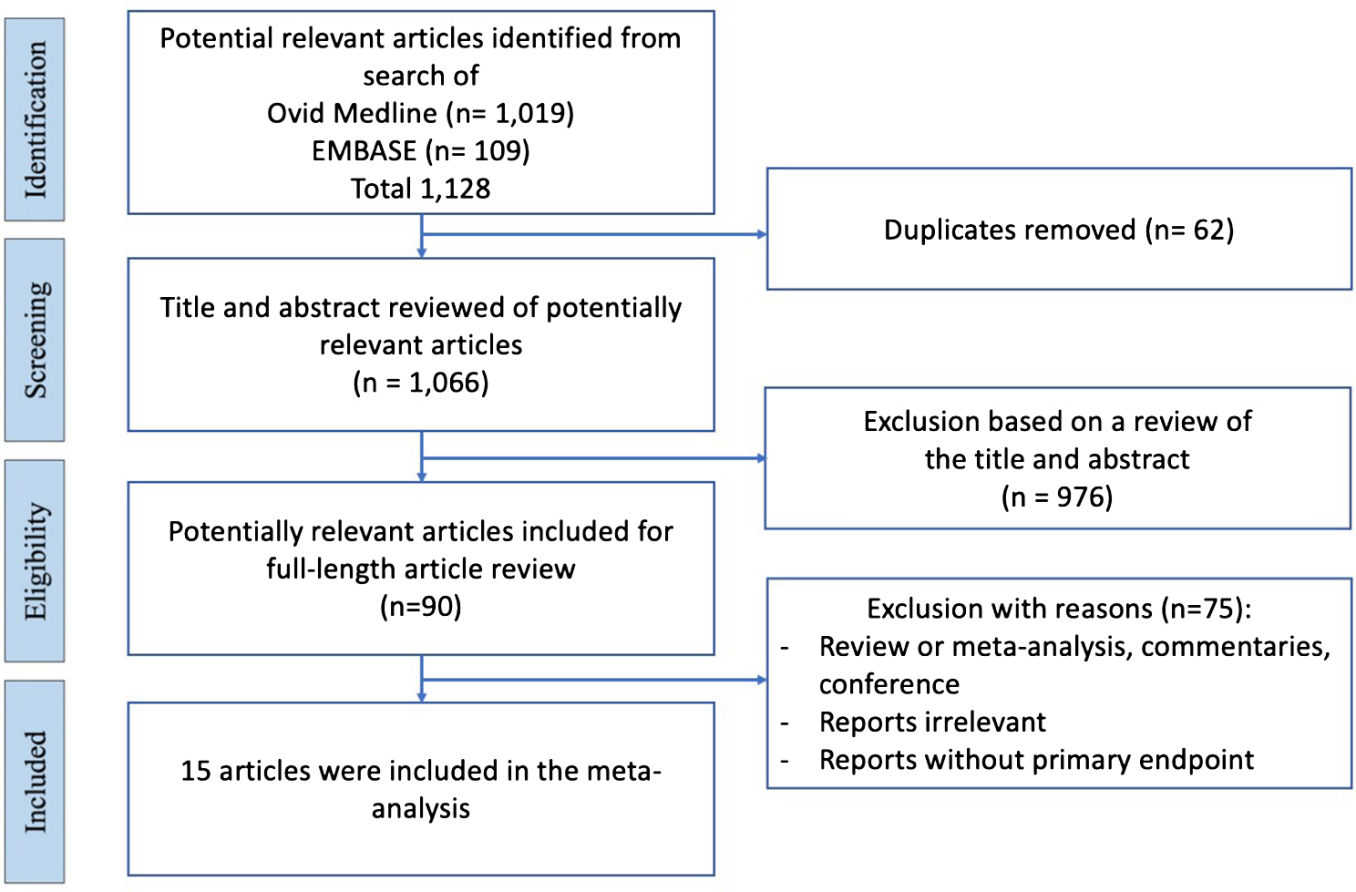
Flow diagram of the article selection procedure based on the PRISMA guideline

We identified 1,128 unique citations (Fig. 1) and reviewed the full text of 90 studies to ascertain eligibility. Fifteen studies (628 patients) were included in this systematic review. There were 2 randomized control trials [9, 10], 5 retrospective cohort studies [11–15], 2 prospective experimental studies [16, 17], and 6 retrospective experimental studies [18–23].

The study characteristics are listed in Table 1. The included studies consisted of postoperative abdominal surgery patients (3 studies) [11, 14, 21], postoperative cardiothoracic surgery patients (5 studies) [10, 13, 19, 20, 22], trauma patients (4 studies) [9, 15, 16, 18], and mixed postoperative and trauma patients (2 studies) [12, 17], and burn patients (1 study) [23]. The prone duration ranged from 4 to 18 h per session. The studies conducted duration of prone position less than 12 h were 6 studies [9, 10, 12, 13, 16, 17], while the studies conducted duration of prone per session with 12 h or greater were 9 studies [11, 14, 15, 18–23].

**Table 1:** Study characteristics

Study/year	Country	Total number Interventions/Controls	Cochrane risk-of-bias tool for randomized trials (ROBINS-I)	Participants	Mean PF ratio at the entry of study	Prone duration per session/ Total prone time/ Number of sessions (h)
**Retrospective cohort study**						
Erhard 1998	Germany	47 28/19	Moderate	Trauma	Unknown	13 [10–15] h/session; 6.1 [1–22] total prone days
Eremenko 2000	Russia	72 36/36	Moderate	ARDS after cardiac syndrome X	Unknown	4–12 h/session; not mentioned total prone day
Davis 2007	USA	61 17/48	Serious	Trauma and general surgery	149 ± 8	3.25 h/session; 6 times/day for 5 days
Gaudry 2017	France	98 36/62	Moderate	Recent abdominal surgery (< 7 days)	91 ± 39	Mean total first prone 15.8 ± 10.4 h; second session 19.2 ± 10.3 h; 1 [1–2] sessions
Akatsuka 2020	Japan	51 24/27	Serious	Abdominal surgery	118 ± 41	16 h/session; 1.5 ± 0.5 times
**Prospective randomized control trial**						
Watanabe 2002	Japan	16 8/8	High	Post transthoracic esophagectomy	166.0 ± 24.9	6 h/session; not mentioned total prone day
Voggenreiter 2005	Germany	40 21/19	High	Multiple trauma patient with ISS at least 16	107 ± 42	11 ± 5 h/session; mean of 7 ± 4 times
**Prospective experimental study**						
Voggenreiter 1999	Germany	22	Moderate	Multiple trauma with blunt chest trauma	Unknown	8 h/session; 9.0 ± 1.12 sessions
Johannigman 2000	USA	20	Moderate	Trauma	148 ± 30	10.3 ± 1.2 h/session; maximum 6 sessions
**Retrospective experimental study**						
Fridrich 1996	Austria	20	Moderate	All trauma-induced ARDS	126.4 ± 8.6	20 h/session; total mean 8 ± 4 days
Johannigman 2001	USA	16	Moderate	Trauma and postoperative surgery	165 ± 18	6.3 ± 1 h/session; 4 sessions
Maillet 2008	France	16	Moderate	Postoperative cardiac surgery	87 ± 26	18 h/session (range, 14–27); not mentioned total prone day
Hale 2012	USA	18	Moderate	Burn	87 ± 37	16 h/session; total 3 [1–6]
Wardenberg 2016	Germany	127	Moderate	Postoperative cardiac surgery	115 ± 46	12 h/session; not mentioned total prone day
Hernández-López 2019	Mexico	7	Moderate	All postoperative patients	83.93 ± 19.12	Total prone duration 57.2 ± 17.2 h (total 3 days)
Saha 2020	Germany	24	Moderate	Postoperative cardiac surgery	Unknown	12 h [12–16] h/session; not mentioned total prone day

### Effects of intervention

#### Primary outcome

**Fig. 2:**
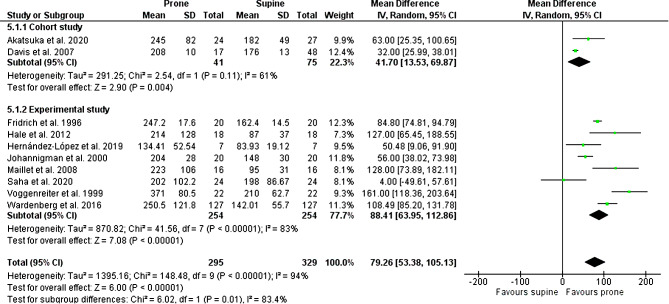
Effect of prone positioning on P/F ratio *P/F ratio, the ratio of arterial oxygen partial pressure to fractional inspired oxygen*

A pooled analysis was performed on the 16 studies using a random effects model. The prone position showed a significantly improved PF ratio compared with the supine position. The mean difference was 79.26 (95% CI, 53.38 to 105.13; I^2^, 94%) (Fig. 2**).** This significant improvement was also demonstrated in both subgroups based on the quality and type of studies. The cohort study subgroup that examined patients in a prone position compared to those who were not prone showed a significantly improved PF ratio (41.70; 95% CI, 13.53 to 69.87; I^2^, 61%). Similarly, there was a significant increase in the PF ratio following proning in the experimental subgroup that compared the patients’ positions in supine and prone (88.41; 95% CI, 63.95 to 112.86; I^2^, 83%).

#### Secondary outcomes

**Fig. 3:**
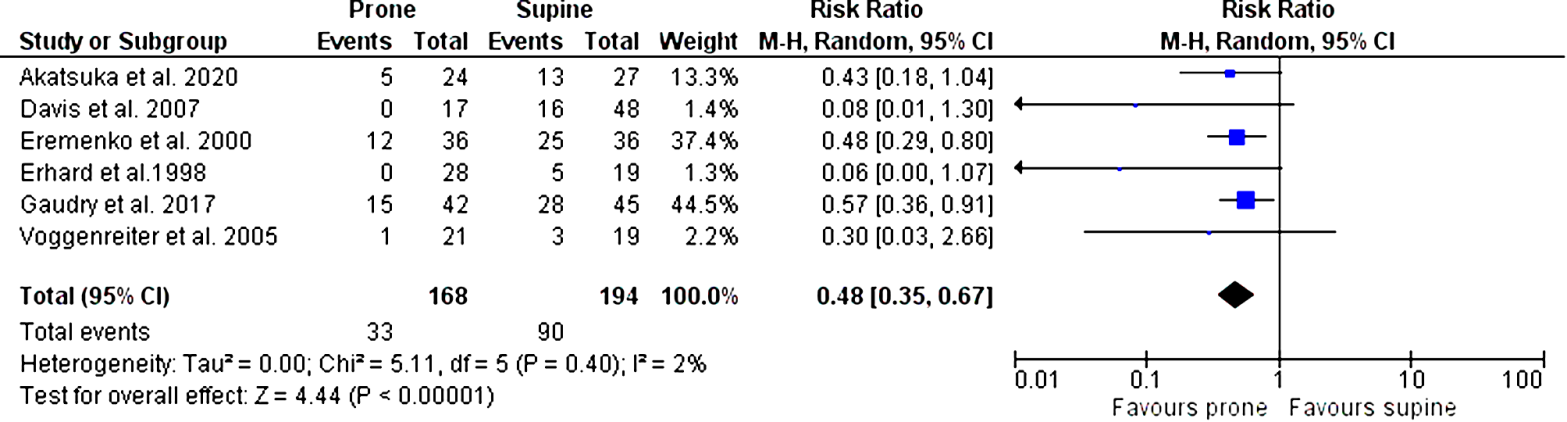
Effect of prone positioning on mortality

Six studies reported in-hospital mortality. There was a significant difference in the mortality rates of the prone and supine positions (RR, 0.48; 95% CI, 0.35 to 0.67; I^2^, 2%; Fig. 3).

**Fig. 4:**
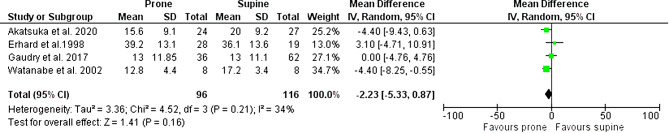
Effect of prone positioning on intensive care unit length of stay (ICU LOS)

**Fig. 5:**
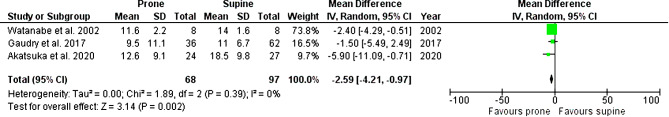
Effect of prone positioning on mechanical ventilator day

The prone position demonstrated no significant difference in ICU length of stay compared with the supine position (-2.23; 95% CI, -5.33 to 0.87; I^2^, 34%; Fig. 4). However, the prone position significantly decreased the mechanical ventilator days (-2.59; 95% CI, -4.21 to -0.97; I, 0%; Fig. 5).

### Publication bias

**Fig. 6:**
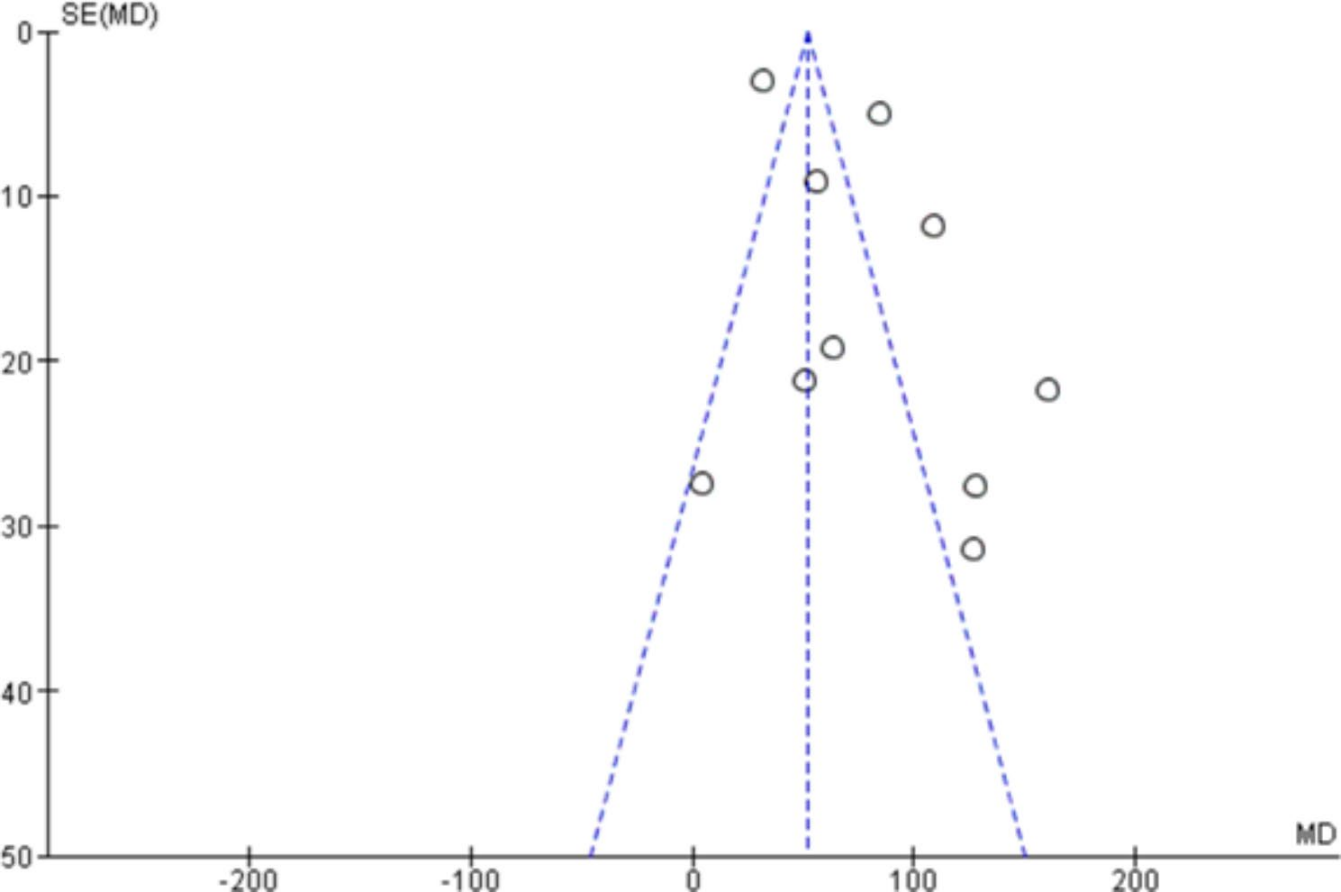
Funnel plot for the meta-analyses of the odds ratio in PF ratio outcome of the prone and supine position groups

The funnel plot of the PF ratio outcome of the prone and supine position groups was relatively symmetric and showed no publication bias (Fig. 6).

### Other effects of prone position

#### Prone position and hemodynamic change

There were limited data on hemodynamic change. We identified 3 studies designed to examine hemodynamic measurements [17, 18, 21]. In general, pulmonary venous catheter with thermodilution technique was utilized for this purpose. Overall, intrapulmonary shunt (Qs/Qt) showed significant reduction in prone position compared to supine position in all studies (*p* < 0.05).

In Hernández‑López et al., the reduction of Qs/Qt demonstrated significantly 48 h after prone positioning until the end of measurement at 72 h. Concomitantly to Fridrich et al., Qs/Qt reductions were observed immediately after prone positioning which showed significant change compared to supine position 20 h after the turn. However, this result demonstrated persistently in the first three cycles, while there was no longer improvement after 72 h.

Johannigman et al. demonstrated significant increase of mean pulmonary artery pressure (mPAP) and pulmonary capillary wedge pressure (PCWP) on days 1 through 4 during prone positioning (*p* < 0.05), while Fridrich et al. reported slow decrease of mPAP and pulmonary vascular resistance (PVR) in the first four days of recording without consistent relation to the turning maneuvers. In addition, cardiac index showed no significant difference between two positions [17, 18].

Three of studies reported cardiac complications (brady-tachy arrhythmias and hemodynamic instability) during prone position [9, 18, 19]. In Voggenreiter 2005 et al., 8 of 19 (42%) patients who underwent prone position reported brady-tachy arrhythmia events, while 2 of 127 (1.54%) in Maillet et al. study reported atrial fibrillation required cardioversion. In Fridrich et al., 6 of 20 (30%) patients were terminated prone positioning due to hemodynamic instability.

#### Prone position and complications

Most studies reported on minor complications, including facial and neck swelling as well as pressure sores [9–11, 15–17, 19, 21, 23]. Three studies reported that all patients undergoing prone positioning developed facial or neck edema [10, 16, 17]. However, this complication was a transient event and was reported to be self-limited.

Surgical wound complications after prone positioning ranged from 3–42% [14, 19, 22]. In cardiac surgery, superficial sternal wound infection ranged from 3–13% [19, 22]. In abdominal surgery patients, Gaudry et al., reported 42% of patients with prone position had surgical wound complications: 8.3% with scar dehiscence, 14% with wound necrosis and leakage, and 3% with abdominal compartment syndrome. However, these rates were not significant different between prone and supine position.

In terms of safety, from 16 studies, 1 of 20 (5%) naso-gastric tube displacement [17], 8 of 20 (40%) peripheral intravenous catheter loss [17], 1 of 20 (5%) central venous catheter loss [18] and 1 event of endotracheal tube loss during prone positioning [18].

## Discussion

This is the first meta-analysis focusing on the effect of prone position in surgical and trauma patients. Our study demonstrated the significant improvement in P/F ratio after proning. This benefit was shown in both overall and subgroup analysis. Prone position in surgical and trauma patients also significantly decreased mortality and mechanical ventilator days. There was no effect on cardiac index regarding two studies. Despite a low rate of serious complications from prone positioning in our systematic review, minor complications, particularly facial edema, were frequently reported among all studies.

Ventilator-induced lung injury (VILI) associated with barotrauma, volutrauma, atelectrauma, and biotrauma demonstrated largely influenced to mortality on ARDS [24, 25]. Lung-protective mechanical ventilation (MV) strategies have demonstrated improved survival in patients with ARDS over the past decades [26]. However, adjusting mechanical ventilation alone may not successfully improve outcomes, including oxygenation, MV day, ICU length of stay and mortality. Consequently, there are alternative methods proposed to help improve outcomes, including prone positioning [27].

One of the current therapeutic strategies for ARDS is prone positioning (PP), which has been studied in numerous major randomized controlled trials (RCT) to obtain the aforementioned benefits [3–5, 28–30]. There are, nevertheless, certain restrictions that were deemed to be proning contraindications. It was unclear if we could execute proning in patients with unstable fractures or unstable hemodynamics due to the shift in position. Therefore, the PROSEVA study’s exclusion criteria have often been implemented as a general contraindication, particularly for patients who have just undergone surgery [4]. However, there are a few relative contraindications to prone positioning that should be chosen on an individual basis. These include hemodynamic instability, trauma-related injuries (such as open abdominal wounds, increased intracranial pressure, unstable long bone or pelvic fracture), and late-term pregnancy. The only absolute contraindication to prone positioning is an unstable spinal fracture [31].

In our study, there was significant oxygenation improvement (increased P/F ratio) in patients who underwent PP compared to supine positioning (SP), (mean difference 79.26; 95% CI, 53.38-105.13; 10 studies; n = 624 patients). This outcome is consistent with the prior meta-analysis that comprised ten randomized control trials comparing supine and prone positioning in ARDS patients undergoing mechanical ventilation. They showed that, within the first three days following randomization, the PF ratio increased by 25–36%, indicating that oxygenation improvements were higher in the prone group than in the supine group [32].

Mortality benefit remained controversial among the studies. According to Sud et al., PP during mechanical ventilation decreased mortality among ARDS patients. The number needed to treat to save one life was 11(95% CI 6–50) [32]. One systematic review and meta-analysis included eight RCTs and evaluated the effect of prone positioning on 28-day mortality. It demonstrated a non-significant reduction in mortality in favour of PP; however, a subgroup analysis of patients with ≥ 12 h of PP found a significantly lower mortality in this group [31]. This mortality reduction was most marked among patients with moderate to severe ARDS. In our study, mortality was significantly improved in PP, (RR 0.48, 95% CI, 0.35–0.67; 6 studies, n = 362), although our included studies were heterogeneous in terms of the duration of PP, varying between < 12 h (6 studies) and ≥ 12 h (9 studies).

The PROSEVA study showed a significant reduction in 28 and 90 days on MV (14 ± 9 days and 33 ± 34 days, respectively) [5]. As in PROSEVA trial, our results indicated a significant reduction in MV days days in PP (mean difference − 2.59, 95% CI, -4.21-0.97; 3 studies; n = 165). While ICU LOS in PROSEVA study showed a trend in favour of PP (*P* = 0.05) ICU LOS, similar to our study demonstrated no significant difference between two groups (mean difference − 2.23, 95% CI, -5.33-0.87; 4 studies, n = 212).

In terms of hemodynamic aspect, Jozwiak et al. demonstrated that the microcirculatory effect of prone position result from three basic mechanisms: an increase in intraabdominal pressure, improvement in arterial oxygenation, and lung recruitment. These three effects can lead to significant increase in cardiac preload, decrease right ventricular afterload, and increase in left ventricular preload. However, cardiac output will increase only in preload reserve patients. While our study did not show increased cardiac output, this result might be explained by the lack of preload assessment data. However, other hemodynamic parameters, especially the decrease of pulmonary vascular resistance and the improvement of intrapulmonary shunt were similarly demonstrated.

Complications from prone positioning must also be noted, particularly since trauma and surgical patients may be at increased risk for morbidity from position changes due to the presence of fractures, surgical incisions, or increased support lines and devices. Adverse events such as facial swelling, loss of venous access, device displacement and pressure sore can occur during transition to and from prone position and during prone positioning itself; however, they can be attenuated with program training. It has been suggested that endotracheal tube obstruction and vasopressor requirement increased with prone position, while the incidence of barotrauma and ventilator-associated pneumonia and unexplained central catheter or endotracheal tube removal were not significant different between groups [33].

In this study, we reported surgical complications that ranged from 3 to 42%, particularly involving abdominal and sternal wound dehiscences related to prone position; however, it was not significantly different compared to supine position. In addition, there were some studies reported the incidence of intraabdominal hypertension or abdominal compartment syndrome in our study. Though prone position can increase intraabdominal pressure, the effect is small. There was no previous reported significant increased intraabdominal pressure resulting in abdominal compartment after prone position [34–36]. The possible explanation are the improper location of the cushion with abdominal compression, and the undetected preexisting intraabdominal hypertension.

Although this is the first meta-analysis of a large number of surgical and trauma patients with ARDS and prone positioning, we must acknowledge the limitations of this study. These include clinical and methodologic heterogeneity among included studies. We attempted to correct for some of these differences via subgroup analyses, but the possibility of bias from the data heterogeneity must be considered. Moreover, our study was unable to assess ARDS severity, especially the individual PF ratio. It is restricted to summarizing the impact of PP with respect to their level of severity. Thus, a large multicenter prospective study is warranted.

In summary, prone position can significantly improve the P/F ratio and has a mortality benefit among surgical and trauma patients who developed acute respiratory distress syndrome. It can cause minor complications, such as facial edema. There was no significant difference in local wound complications compared to those with supine position. Prone position may be an effective rescue therapy for surgical and trauma patients.

## Data Availability

All data analysed during this study are included in this published article.

## Declarations

### Presentation

This study was presented as an ePoster at the *Critical Care Canada Forum 2022 Conference* in Toronto, ON on November 22–25, 2022.

### Implication statement

It is safe to apply prone position as a rescue therapy in trauma or surgical patients who are diagnosed with acute respiratory distress syndrome. Prone position can improve oxygenation without significant major complications.
